# Strengthening of Full-Scale Laminated Veneer Lumber Beams with CFRP Sheets

**DOI:** 10.3390/ma15196526

**Published:** 2022-09-20

**Authors:** Michał Marcin Bakalarz, Paweł Grzegorz Kossakowski

**Affiliations:** Faculty of Civil Engineering and Architecture, Kielce University of Technology, 25-314 Kielce, Poland

**Keywords:** composites, carbon fiber, load-bearing capacity, reinforcement, stiffness, wood structures

## Abstract

This paper presents the results of experimental research on full-size laminated veneer lumber (LVL) beams unreinforced and reinforced with CFRP sheets. The nominal dimensions of the tested beams were 45 mm × 200 mm × 3400 mm. The beams were reinforced using the so-called U-type reinforcement in three configurations, differing from each other in the thickness of the reinforcement and the side surface coverage. An epoxy resin adhesive was used to bond all the components together. A four-point static bending test was performed according to the guidelines in the relevant European standards. The effectiveness of the reinforcement increased with the level of coverage of the side surface and the level of reinforcement. The average increases of bending resistance were 42%, 51% and 58% for configurations B, C and D, respectively. The average value of bending stiffness increased for the beams of series B, C and D by 15%, 31% and 43%, respectively. Their failure mode changed from brittle fracture initiated in the tensile zone for unreinforced beams to more ductile fracture, initiated in the compression zone. The influence of the coverage of the side surface by the CFRP sheet and reinforcement ratio on the mechanism of failure and effectiveness of strengthening was studied in the article.

## 1. Introduction

The reinforcement of wooden structures is still a significant problem from scientific and practical points of view. Combinations of two or more materials provide composites with different or better properties than those of the components when used separately. This makes it possible to optimize structural components and adapt their strength to suit the expected loads.

A commonly used technique to reinforce or repair bent wooden (solid or glued) beams is the introduction of additional elements withing existing structures [1,2]. The additional elements may include bars [3,4,5,6,7,8], laminates [9,10,11,12,13], meshes [14] or sheets [15]. These elements may be made of steel [16] or composites reinforced with carbon [17,18,19], glass, basalt and aramid fibers. The reinforcing elements may be installed externally or internally, in the tensile and/or compression zone of the beam.

### 1.1. State of the Art of Use a FRP Sheets as Reinforcement

Sheets for strengthening building structures are produced in the form of un-, bi- or multi-directionally reinforced elements which have been impregnated with an adhesive prior to their application. Aramid, basalt, glass and carbon fibers are used commonly as reinforcements. Sheets are characterized by their low weight, high strength and elasticity. 

The following types of strengthening configurations for composite sheets can be distinguished:

- sheets applied in the form of a strip which is bonded to the bottom surface of the element or applied along the bottom surface in the middle of the beam with their ends anchored on the sides [20,21,22] or the top surface;
- sheets bonded to the bottom and side surfaces with varying coverage of the latter, i.e., so-called U-type reinforcements;
- sheets bonded vertically or horizontally inside the cross-section;
- sheets completely covering the test piece, i.e., wrapping all sides with the FRP composite.

The correlation between the number of CFRP sheet layers and the load bearing capacity of the beams was studied at a laboratory scale by Li et al. [23]. It was shown that one layer was enough to increase the maximum loading capacity by 40%. Kossakowski [24] analyzed the load bearing capacity of solid timber beams reinforced with aramid, glass and carbon sheets. Meanwhile, a comparison of the effectiveness of uni- and bi-directional reinforcements using CFRP and BFRP sheets of different weights in a U-type reinforcement arrangement was described by De la Rosa García et al. [13]. The possibility of the commercial use of fast-growing, cheaper species of wood by improving its properties through the use of internal reinforcements, i.e., GFRP sheets bonded vertically, was discussed by Basterra et al. [25]. In that study, the reinforcement resulted in a 23% increase in the maximum bending moment. Gezer and Aydemir [26] observed a significant increase in compressive strength parallel to the grain and bending strength for reinforced beams by wrapping the beams with a sheet that had been reinforced with carbon fiber. Gómez et al. [27] presented a comparative study of the behavior of wooden beams reinforced with four types of unidirectionally reinforced FRP fabrics and metal fibers. Meanwhile, the use of a unidirectional reinforcement consisting of CFRP sheets bonded from below as a passive reinforcement of glued laminated timber beams was analyzed by Vahedian et al. [28]. Yang et al. [29] studied the behavior of 100 × 150 × 2000 mm wooden beams reinforced with a hybrid composite, i.e., a combination of CFRP and GFRP sheets stacked on top of each other. Donadon et al. [30] analyzed the bending stiffness of glued, laminated timber beams strengthened with a polymer composite reinforced with unidirectional VECTRAN fibres. Finally, Ghanbari-Ghazijahani et al. [31] presented experimental and numerical research on the reinforcement of light I-section beams with flanges made of laminated veneer lumber and a web made of OSB boards. 

### 1.2. Objectives

The use of composite materials as passive reinforcements of structural elements made of wood, as well as the engineering of wood products subjected to flexion, have been studied by many researchers. However, few studies have attempted to strengthen laminated veneer lumber beams. The reason for this could be the high cost and relatively short lifespan of LVL structural elements in civil engineering settings. Nevertheless, as LVL is a wood-based composite, its deterioration and damage may be caused by many factors including the influence of the external environment, human errors, or increasing loads over time. Therefore, it is to be expected that in future, the necessity to strengthen structures made of LVL will become increasingly apparent.

This study investigates the behavior of laminated veneer lumber beams strengthened with carbon fiber-reinforced polymer sheet bonded to the external faces with epoxy resin and subjected to bending under static loads. The influence of the degree of coverage of the side surface by the composite and the reinforcement ratio on load bearing capacity, bending stiffness and the mechanism of failure of the strengthened, full scale LVL beams is discussed in detail. 

The presented strengthening technique is effective, easy and rapid. However, as it covers almost the entire external surface of strengthened beams, it may negatively affect the aesthetics of the latter.

## 2. Materials and Methods

### 2.1. Materials

#### 2.1.1. Laminated Veneer Lumber (LVL)

Tests were carried out on full-size laminated veneer lumber (LVL) beams made of pine and spruce veneers. Each beam consisted of 15 layers of veneer, each of which was approximately 3 mm thick. The nominal dimensions of the beams tested were 45 mm × 200 mm × 3400 mm. The properties of the LVL beams are given in Table 1.

#### 2.1.2. CFRP Sheets

Unidirectional carbon fiber reinforced polymer (CFRP) sheets with a thickness of 0.333 mm and a weight of 600 g/m^2^ were used as a reinforcement. The sheets were delivered in rolls with a width of 300 mm. Then, they were cut with scissors into strips of 2800 mm in length. The properties of the CFRP sheet are given in Table 2.

#### 2.1.3. Adhesive

For lamination, S&P Resin 55 HP epoxy resin was used. The adhesive was applied to the composite sheet surface and the veneer surface. Prior to applying the adhesive, the veneer surface was sanded, and the edges of the beams were rounded. The radius on the corners was 6.25 mm. The composite sheet was properly cleaned before applying the adhesive. Selected properties of epoxy resin are given in Table 3.

### 2.2. Methods

**Figure 1:**
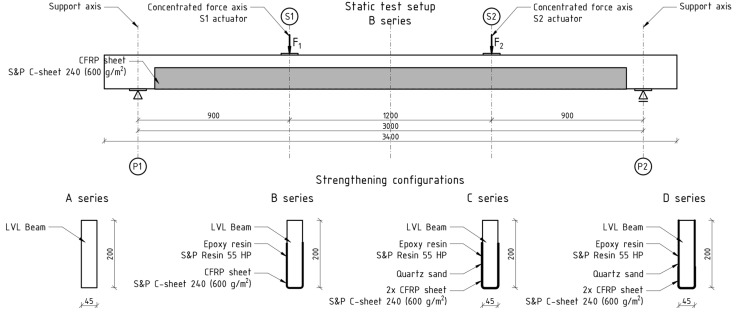
Strengthening configurations and schematic of the test setup.

The tests were carried out in the Material Strength Laboratory of the Kielce University of Technology. The aim of the study was to analyze the effectiveness of the aforementioned reinforcement technique. The scope of the research included the preparation of four series of five elements, according to the following scheme (Figure 1):

- A series—unreinforced beams;
- B series—beams reinforced with one layer of CFRP sheet bonded to the bottom side (reinforcement ratio, *ρ_t_* = 1.11%);
- C series—beams reinforced with two layers of CFRP sheet bonded to the (reinforcement ratio, *ρ_t_* = 2.22%);
- D series—beams reinforced with two layers of CFRP sheet covering entire side surfaces with overlap in the tension zone (reinforcement ratio of tensile zone *ρ_t_* = 1.48%; reinforcement ratio of compression zone *ρ_c_* = 0.74%).

**Figure 2:**
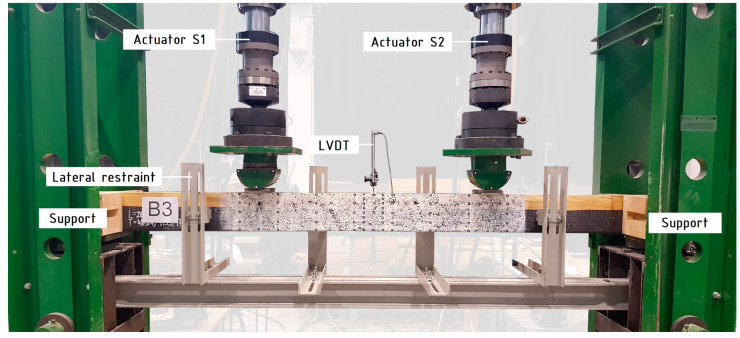
Image of the test setup.

The bending tests were carried out in accordance with the requirements of the standards [34,35] i.e., four-point static bending tests. The beams were loaded with two concentrated forces applied to their upper surface. The distance from the support axis to the axis of the nearest concentrated force was 900 mm. The axial spacing of the supports was 3000 mm and the concentrated forces 1200 mm. One hundred millimeter-wide steel plates were used on the supports and in places where the concentrated forces were applied. Additional supports to prevent displacements from the bending plane were used at a distance of about 300 mm from the axis of the supports, at the front and rear of the beam. A photo of the test stand is shown in Figure 2.

The loading force *F*, the beam deflection *u* and the duration of the test *t* were continuously recorded. *u* was measured using an inductive sensor in the middle of the beam span, at the level of the outermost compressed fibers. *F* is the sum of the forces imposed by the *S*1 and *S*2 actuators. After the tests, the failure mode was recorded and the laminated veneer lumber moisture content was verified using a Tanel WRD-100 moisture meter. The average value of LVL moisture content in each series was approximately equal (Table 4).

## 3. Results and Discussion

### 3.1. Bending Behavior and Load Bearing Capacity

**Figure 3:**
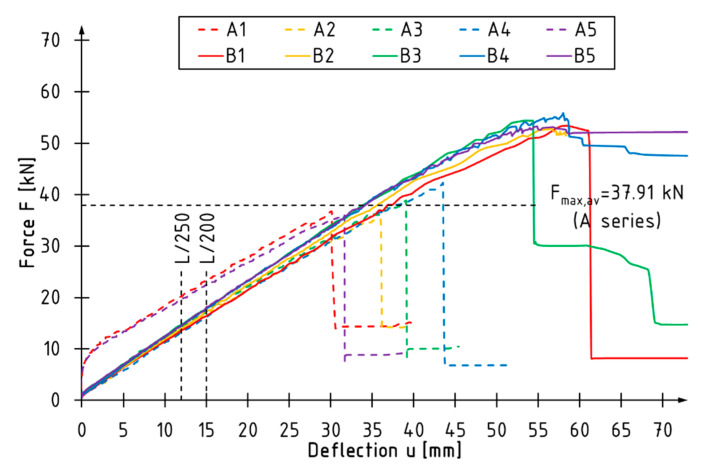
Load-deflection curves of B series beams.

**Figure 4:**
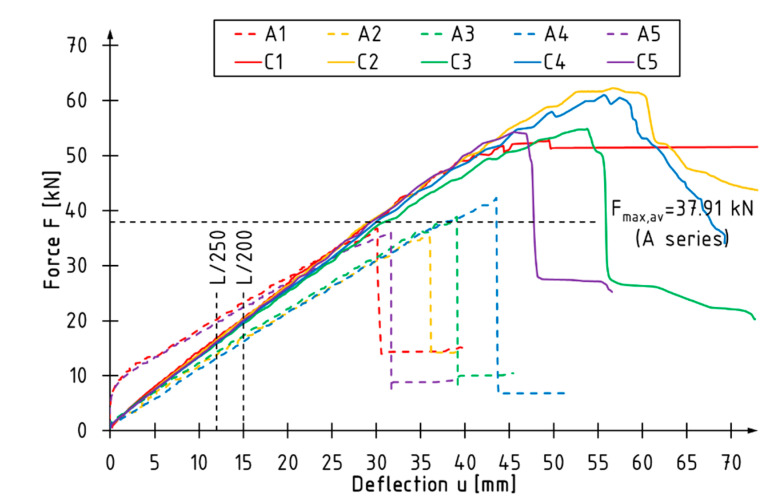
Load-deflection curves of C series beams.

**Figure 5:**
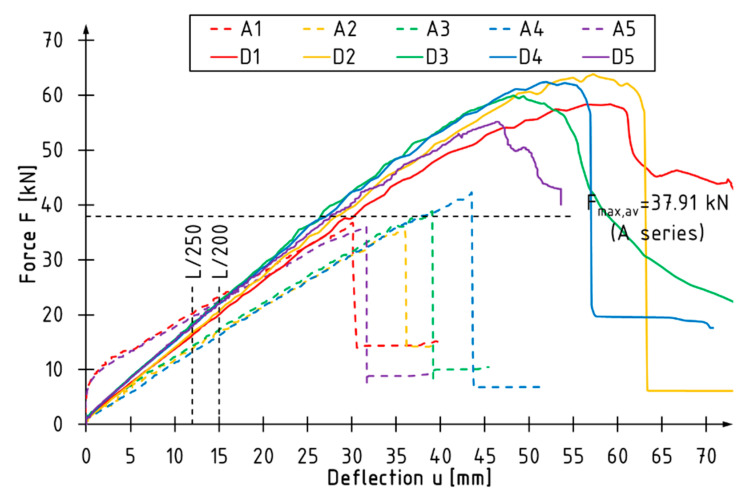
Load-deflection curves of D series beams.

Figure 3, Figure 4 and Figure 5 present load vs. deflection diagrams for all tested beams. The vertical lines indicate the limit deflection values adopted according to standard [36]. The horizontal line marks the average value of the maximum loading force for the A series beams. Each series of reinforced beams is shown in a separate diagram. Unreinforced beams are plotted with dashed lines.

The unreinforced beams showed linear behavior from the beginning of the test to the point of failure. In the case of the B series beams, the curves describing the dependence of the loading force on the deflection almost coincided, and the failure occurred at a similar level of loading force (Figure 3). The curves of the beams reinforced with two layers of CFRP sheets (Series C and D) had similar characteristics in terms of the linear-elastic range (Figure 4 and Figure 5). For reinforced beams, more ductile behavior was observed in the final phase of the test compared to the reference beams. As the level of reinforcement increased, the utilization of the compression zone and bending ductility increased.

The results of the experimental tests, describing the maximum loading force *F_max_*, maximum bending moment *M_max_* and deflection at maximum force *u_F,max_*, are shown in Table 5, Table 6, Table 7 and Table 8; the percentage increment values in relation to the reference beams are shown in brackets. Additionally, the beam failure modes are given in the table and are discussed in detail below.

The percentage increase in the load bearing capacity was observed with the increase of the thickness of reinforcement and the coverage of the side surface with the composite. The average value of the maximum loading force was 37.91 kN for the reference beams and 53.96 kN, 57.13 kN, 59.97 kN for the B, C and D series beams, respectively. Reinforcement with one sheet layer contributed to a >40% increase in the load bearing capacity, while reinforcement with two layers contributed to a >50% increase. It should be noted that with the use of the same number of carbon sheets (C and D series beams), it turned out to be more effective to cover the entire side surfaces with the reinforcement overlap in the tensile zone. 

The inverse relationship was observed for the deflection recorded at maximum load—the higher the level of the reinforcement, the lower the value of deflection at failure. This phenomenon is related to the increase in bending stiffness, which contributed to earlier failure initiation. 

### 3.2. Bending Stiffness

The bending stiffness of beams *k* was estimated on the basis of the slope of the linear-elastic part of the load-deflection curves, as shown in Figure 3, Figure 4 and Figure 5. The stiffness coefficient of the beam was determined according to the formula:

(1) $$k=\frac{\Delta F}{\Delta u},$$

where Δ*F* is the change of the loading force [kN] and Δ*u* is the deflection corresponding to the change of the loading force [mm].

The average values of the bending stiffness coefficient are presented in Table 9. The largest increase was recorded for the D series beams and the smallest for the B series beams.

### 3.3. Failure Modes

The primary mechanism of failure of the reinforced beams involved the destruction of the laminated veneer lumber in the compression zone. In the case of the B series beams, the rupture of the CFRP sheet in the tension zone was also recorded (one layer of reinforcement), due to the insufficient reinforcement ratio. For C and D series, the CFRP sheets were not destroyed in the tension zone, but were damaged in the compression zone due to the movement of destroyed veneers. 

**Figure 6:**
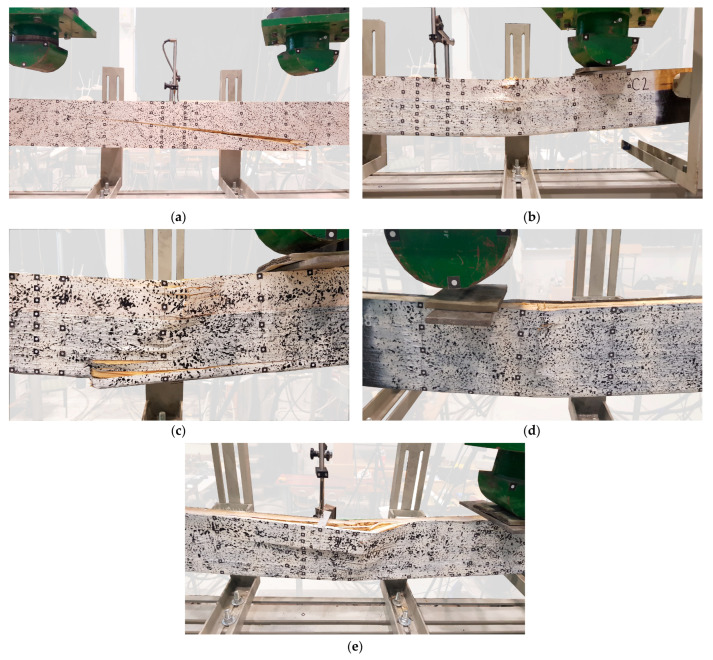
Failure modes: (**a**) tension (beam A4); (**b**) compression (beam C2); (**c**) compression + tension + rupture of CFRP sheet (beam B3); (**d**) shear of composite sheet + compression (beam D3); (**e**) compression + sheet delamination (beam D2).

Recorded failure modes for tested beams:

- tension—failure of the reference beams resulted from the cracking of the veneer, which initiated in the outermost fibers stretched between the concentrated force points in the zone of maximum bending; this was also observed for weakly reinforced beams (see Figure 6a);
- compression—this is typical for elements reinforced with fibrous composites, with the following consequences: local buckling of the veneer layers and delamination; shearing and the accompanying displacement of the adjacent sections of the beam, or the penetration of one part of the veneer into another (see Figure 6b–e);
- delamination of the sheets perpendicular to the main direction; this usually occurs in the compressed zone and is associated with the breakage of the transversally arranged polyester fibers that stabilize the fibers arranged in the main direction (see Figure 6e);
- sheet shearing—i.e., carbon fiber cuts (perpendicular to the main direction of the sheet), related to the overlapping of adjacent veneer sections at the point of the failure initiation (Figure 6d);
- sheet delamination—breaks in the continuity of carbon fibers in the tensile zone, as observed during the tests of the B series beams (Figure 6b);
- lateral torsional buckling—a loss of stability.

### 3.4. Analysis of Modulus of Rupture

**Figure 7:**
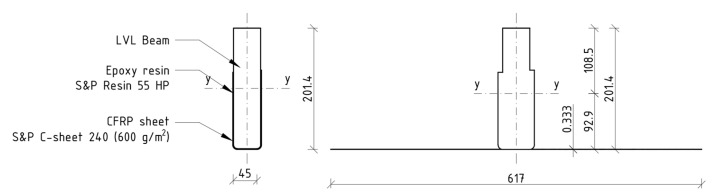
Transformed cross section of beam B3

A transformed cross section method was applied to define the mechanical model of laminated veneer lumber beams with CFRP sheets; linear distribution of stress across the depth of the cross section was assumed. The moduli of elasticity values, i.e., 240 GPa and 14 GPa for CFRP sheet and LVL respectively, were taken from experimental tests (laminated veneer lumber) and manufacturer data sheets (CFRP sheet). Figure 7 shows an example of a transformed cross section of a B series beam.

An assumed mechanical model was used to predict the bending strength of laminated veneer lumber beams with CFRP sheets. Bending strength (Modulus of Rupture—*MOR*), defined as the maximum bending stress at failure, was evaluated according to the formula:

(2) $$MOR=\frac{M_{max}\;z}{I_{y}},$$

where:

*M_max_* is the maximum bending moment recorded during experimental tests [kNm]; *z* is the distance between the neutral axis and external compressed fibres [m]; and *I_y_* is the moment of inertia of transformed cross section [m^4^].

The results of our analysis are given in Table 10. It was found that the moment of inertia of the transformed cross section increased with an increase of the reinforcement ratio. The application of CFRP sheets in the compression and tension zones (D series) reduced the vertical displacement of the neutral axis in comparison with the application of the same number of CFRP sheets in the tension zone only (C series). This approach reduced the average modulus of rupture values for beams series C and D in comparison to series B. 

## 4. Conclusions

The paper presents the results of experimental research on the reinforcement of laminated veneer lumber beams with CFRP sheets bonded to external surfaces with an epoxy resin adhesive. The influence of the reinforcement on the bearing capacity and bending stiffness, as well as the failure mode of the beams, is described in detail. It was found that:

1. The results of experimental research confirmed the validity of using fiber composites in the form of sheets as reinforcements for simply-supported LVL beams.
2. The thicker the coverage of the side surface, the higher the load capacity of the beam. In the ultimate limit state of the load capacity, the highest percentage increase of load at failure was 58% for the D series beams reinforced with two layers of CFRP sheets covering the entire side surface of the beams. The use of two layers of sheets in the tensile zone only (C series) contributed to a 51% increase in the maximum load, while for one layer of CFRP sheet, the increase was 42%.
3. CFRP sheets achieve superior reinforcement of LVL beam compared to CFRP laminate bonded to the bottom surface [37] or bonded into predrilled grooves on the bottom surface [38]. This may be due to the binding of timber defects by covering the side surface.
4. A greater increase in bending strength and stiffness was observed in the full-sized beams than in those tested on a laboratory scale [39,40]. As the volume of the LVL beams increased, their bending strength decreased. CFRP sheets were also found to be more effective than aramid or glass fiber reinforced sheets [41]. Additionally, CFRP sheets have better mechanical properties than to AFRP and GFRP sheets.
5. The highest percentage increase in bending stiffness was observed for the C and D series beams, amounting to 31% and 43%, respectively. The smallest increase, amounting to 15%, was observed for the B series beams.
6. With the increase of the cross-section reinforcement thickness and the side surface thickness, the percentage increase of the deflection at the maximum force in relation to the reference beams decreased. The deflection increment for the B series beams was 42%, and for the D series beams 33%. Failure initiation signs occurred at a lower deflection value.
7. There was a change in the failure mode, i.e., the brittle fracturing observed with the reference beams did not occur. The load-bearing fibers broke in cases where one layer of CFRP sheet was used. Regardless of the number of fabric layers, delamination occurred as a result of breaking the polyester stabilizing fibers in the compressed zone.
8. The use of composite sheets as reinforcements is justified to increase safety by eliminating the influence of possible defects in the structure of glued laminated wood with veneers.
9. The proposed reinforcement method is simple and quick to apply. It does not require any special equipment and can be performed by two people.
10. The proposed reinforcement schemes can be used for existing structures.
11. In further tests, the use of additional (structural) reinforcements should be considered to prevent the delamination of the sheets arranged transversely to the main reinforcement direction in the compression zone.

## Figures and Tables

**Table 1:** Selected mechanical and physical properties of LVL [32].

Parameter	Value
Bending strength (edgewise condition) *f_m,_*_0*,edge*_ [MPa]	44
Modulus of elasticity (parallel to grain) *E*_0*,mean*_ [GPa]	14
Tension strength (parallel to grain) *f_t,_*_0,*k*_ [MPa]	36
Compression strength (parallel to grain) *f_c,_*_0,*k*_ [MPa]	40
Mean value of density *ρ_d_* [kg/m^3^]	550

**Table 2:** Material parameters of CFRP sheet [33].

Parameter	Value
Modulus of elasticity *E_f_* [GPa]	265
Tensile strength *f_t,f_* [MPa]	5100
Density *ρ_f_* [kg/m^3^]	1800
Elongation at rupture *ε_f_* [%]	1.7–1.9
Design thickness *t_f_* [mm]	0.333

**Table 3:** Technical data of epoxy resin [33].

Parameter	Value
Modulus of elasticity *E_k_* [MPa]	3200
Density *ρ_k_* [kg/m^3^]	1200–1300
Compressive strength *f_c,k_* [MPa]	100

**Table 4:** Average moisture content value of laminated veneer lumber in each series.

Series	A	B	C	D
Moisture content [%]	14.0	14.1	12.7	13.8

**Table 5:** Series A test results.

Parameter	Beam					Mean Value
	A1	A2	A3	A4	A5	
*F_max_* [kN]	36.87	35.33	38.89	42.36	36.08	37.91 ± 1.90
*M_max_* [kNm]	16.59	15.90	17.50	19.06	16.24	17.06 ± 1.27
*u_max_* [mm]	-	36.06	39.10	43.56	-	39.57 ± 3.77
Failure mode	Tension	Tension	Tension	Tension	Tension	-

**Table 6:** Series B test results.

Parameter	Beam					Mean Value
	B1	B2	B3	B4	B5	
*F_max_* [kN]	53.39	52.83	54.39	55.88	53.27	53.96 ± 1.22 (+42%)
*M_max_* [kNm]	24.03	23.77	24.48	25.15	23.97	24.28 ± 0.55 (+42%)
*u_max_* [mm]	58.20	56.64	53.44	58.07	54.87	56.24 ± 2.07 (+42%)
Failure mode	Compression + tension + rupture of CFRP sheet	Compression (lateral torsional buckling)	Compression + tension + rupture of CFRP sheet	Compression (lateral torsional buckling)	Compression (lateral torsional buckling)	-

**Table 7:** Series C test results.

Parameter	Beam					Mean Value
	C1	C2	C3	C4	C5	
*F_max_* [kN]	53.32	62.21	54.86	61.01	54.27	57.13 ± 4.14 (+51%)
*M_max_* [kNm]	23.99	27.99	24.69	27.45	24.42	25.71 ± 1.86 (+51%)
*u_max_* [mm]	49.93	56.64	53.79	55.60	45.53	52.89 ± 5.05 (+34%)
Failure mode	Compression (lateral torsional buckling)	Compression + delamination of CFRP sheet	Compression + delamination of CFRP sheet	Compression + shear of CFRP sheet	Compression + delamination of CFRP sheet	-

**Table 8:** Series D test results.

Parameter	Beam					Mean Value
	D1	D2	D3	D4	D5	
*F_max_* [kN]	58.33	63.87	59.99	62.48	55.18	59.97 ± 3.43 (+58%)
*M_max_* [kNm]	26.25	28.74	26.99	28.11	24.83	26.98 ± 1.55 (+58%)
*u_max_* [mm]	59.23	57.22	48.24	51.88	46.39	52.59 ± 5.55 (+33%)
Failure mode	Compression + shear of CFRP sheet	Compression + delamination of CFRP sheet	Compression + shear of CFRP sheet	Compression + rupture of CFRP sheet	Compression + delamination of CFRP sheet	-

**Table 9:** Average values of stiffness coefficient *k*.

Parameter	Series			
	A	B	C	D
*k* [kN/mm]	0.900 ± 0.022	1.036 ± 0.046 (+15%)	1.182 ± 0.025 (+31%)	1.283 ± 0.101 (+43%)

**Table 10:** Analytical analysis results.

Parameter	Series			
	A	B	C	D
*z* [m]	0.100	0.108	0.114	0.108
*I_y_* [m^4^]	3.45 × 10^−7^	3.45 × 10^−7^	3.84 × 10^−7^	4.54 × 10^−7^
*MOR* [MPa]	57.83	72.33	68.20	59.97
